# Supplementary material for: Resolving the full spectrum of human genome variation using Linked-Reads
Source: Genome Res. 2019 Apr;29(4):635–45. doi: 10.1101/gr.234443.118 (PMC6442396; doi:10.1101/gr.234443.118)
Supplement: Supplemental Material [file supp_gr.234443.118_Supplemental_longranger-2.2.zip › longranger-2.2.tar.bz2!/lr2.2-public/lib/stan/cmdstan/stan/doc/api/html/classstan_1_1mcmc_1_1fixed__param__sampler-members.html]

Stan: Member List


|  |  |
| --- | --- |
|  | Stan  2.5.0  probability, sampling & optimization |


- Main Page
- Related Pages
- Namespaces
- Classes
- Files

- Class List
- Class Index
- Class Hierarchy
- Class Members

All Classes Namespaces Files Functions Variables Typedefs Enumerations Enumerator Friends Macros Pages

- stan
- mcmc
- fixed\_param\_sampler

stan::mcmc::fixed\_param\_sampler Member List

This is the complete list of members for stan::mcmc::fixed\_param\_sampler, including all inherited members.

|  |  |  |
| --- | --- | --- |
| base\_mcmc(std::ostream \*o, std::ostream \*e) | stan::mcmc::base\_mcmc | inline |
| err\_stream\_ | stan::mcmc::base\_mcmc | protected |
| fixed\_param\_sampler(std::ostream \*o=&std::cout, std::ostream \*e=0) | stan::mcmc::fixed\_param\_sampler | inline |
| get\_sampler\_diagnostic\_names(std::vector< std::string > &model\_names, std::vector< std::string > &names) | stan::mcmc::base\_mcmc | inlinevirtual |
| get\_sampler\_diagnostics(std::vector< double > &values) | stan::mcmc::base\_mcmc | inlinevirtual |
| get\_sampler\_param\_names(std::vector< std::string > &names) | stan::mcmc::base\_mcmc | inlinevirtual |
| get\_sampler\_params(std::vector< double > &values) | stan::mcmc::base\_mcmc | inlinevirtual |
| name() | stan::mcmc::base\_mcmc | inline |
| name\_ | stan::mcmc::base\_mcmc | protected |
| out\_stream\_ | stan::mcmc::base\_mcmc | protected |
| transition(sample &init\_sample) | stan::mcmc::fixed\_param\_sampler | inlinevirtual |
| write\_sampler\_param\_names(std::ostream &o) | stan::mcmc::base\_mcmc | inlinevirtual |
| write\_sampler\_params(std::ostream &o) | stan::mcmc::base\_mcmc | inlinevirtual |
| write\_sampler\_state(std::ostream \*o) | stan::mcmc::base\_mcmc | inlinevirtual |
| ~base\_mcmc() | stan::mcmc::base\_mcmc | inlinevirtual |


---

[ Stan Home Page ]

*© 2011–2014,
Stan Development Team.*
